# Neural network connectivity by optical broadcasting between III-V nanowires

**DOI:** 10.1515/nanoph-2025-0035

**Published:** 2025-07-04

**Authors:** Kristians Draguns, Vidar Flodgren, David Winge, Alfredo Serafini, Aigars Atvars, Janis Alnis, Anders Mikkelsen

**Affiliations:** University of Latvia, Riga, Latvia; 5193 Lund University, Lund, Sweden

**Keywords:** optical neural networks, nanowires, III-V, semiconductors

## Abstract

Biological neural network functionality depends on the vast number of connections between nodes, which can be challenging to implement artificially. One radical solution is to replace physical wiring with broadcasting of signals between the artificial neurons. We explore an implementation of this concept by light emitting/receiving III-V semiconductor nanowire neurons in a quasi-2D waveguide. They broadcast light in anisotropic patterns and specific regions in the nanowires are sensitised to exciting and inhibiting light signals. Weights of connections between nodes can then be tailored using the geometric light absorption/emission patterns. Through detailed simulations, we determine the connection strength based on rotation and separation between the nanowires. Our findings reveal that complex weight distributions are possible, indicating that specific neuron geometric patterns can achieve highly variable connectivity as needed for neural networks. An important design parameter is matching the wavelength to the specific physical dimensions of the network. To demonstrate applicability, we simulate a reservoir neural network using a hexagonal pattern of nanowires with random angular orientations, displaying its ability to perform chaotic time series prediction. The design is compatible with integration on Si substrates and can be extended to other nanophotonic components.

## 1 Introduction

For bio-inspired neural networks, the performance is related to the number and complexity of connections between the neurons. Using electrical connections as in traditional computing technology is a challenge as it can be difficult to fabricate the many connections in a planar geometry and capacitive losses in the wiring can lead to high energy consumption [1], [2], [3], [4]. Using light for the connections allows broadcasting of many signals in the same space, photons in the visible/infrared range carry little energy and transmission is fast [1], [5].

Photonic solutions based on existing technologies, such as SiO_2_ waveguides, have shown considerable progress in recent years for neuromorphic computing [1], [6], [7], [8], [9]. However, their physical footprint is large (governed by the light wavelength) and while energy expenditure can in principle be low, it is limited by losses in optical-electronic conversion of macroscopic components. An important part of the solution to the photon–electron conversion losses is the use of nanostructures for receiving/emitting of the optical signals [4] and multiplexing of signals in the same waveguide can reduce the footprint.

III–V semiconductor nanowires (NWs) represent a mature nanotechnology platform, with an extremely broad range of available structures with record-breaking performance for electronics and photonics [10], [11], [12], [13], [14], [15]. The direct bandgaps available over a large wavelength range makes III-V NWs especially suitable for optoelectronic photodetectors and LEDs. Further their light emission and absorption will be anisotropic, depending on their shape.

Recently it was proposed [16], [17] that III-V NW devices can be used for nodes in a bio-inspired nanophotonic neural network. The evaluation of optical signals is then performed by neuron-like nodes constructed from the NWs. This minimizes power consumption of the network [16]. Communication between such NWs is experimentally realizable as shown recently [18]. Memory can be implemented by introducing e.g. light sensitive molecules in the medium between the NWs [19]. Connectivity between nodes is achieved by broadcasting overlapping light signals inside a shared quasi-2D waveguide (like a mobile network) which dramatically reduce the footprint of the circuit. The differing weights of individual connections were achieved by shaping the light emission and using the wavelength sensitivity of the NWs [16]. However, in the specific network solution, only limited variability in connection weight was needed in this work. Thus, it is still an open question how large variability in connection weights is possible in a nano optoelectronic broadcasting system. Here the fundamental case of using the geometric sub-wavelength variability of light emission and reception of NWs is an interesting starting point as it requires only two nanostructured components that will be identical for all neural nodes.

To evaluate neuromorphic concepts in a functional network, so-called reservoir neural networks are often tested [20], [21], [22]. In these recurrent neural networks (also called echo state networks), the neural nodes connected with random weights (the reservoir) while the input and output weights of the network are trained. Although simple, these networks have a number of interesting applications in e.g. pattern recognition and prediction of signals [20].

In the present work, we explore an optical neural network with nodes based on NW components. The nodes broadcast signals between each other on a single wavelength for both inhibiting and exciting signals that are combined in a sigmoid functional that can result in directional light emission at the same wavelength. To reach a basic understanding of such systems, we use the simplest possible components and geometries in which the node has one receiving and one emitting wire with a circuit footprint of ∼3 μm in diameter. We address the interesting question on how wide a range of connectivity weights are possible using only geometric variations of the component placement. We focus on optical simulations using FDTD of light concentration as the electrical function and photo-electron conversion of the NW neural nodes has been investigated in detail elsewhere [16]. We show that complex weight patterns are achievable between the nodes, even in this simple system. We show by a detailed simulation that our hardware concept can run a reservoir network. The system performance depends strongly on the geometry of the network design in relation to the used wavelength.

## 2 Nanowire neural node

**Figure 1:**
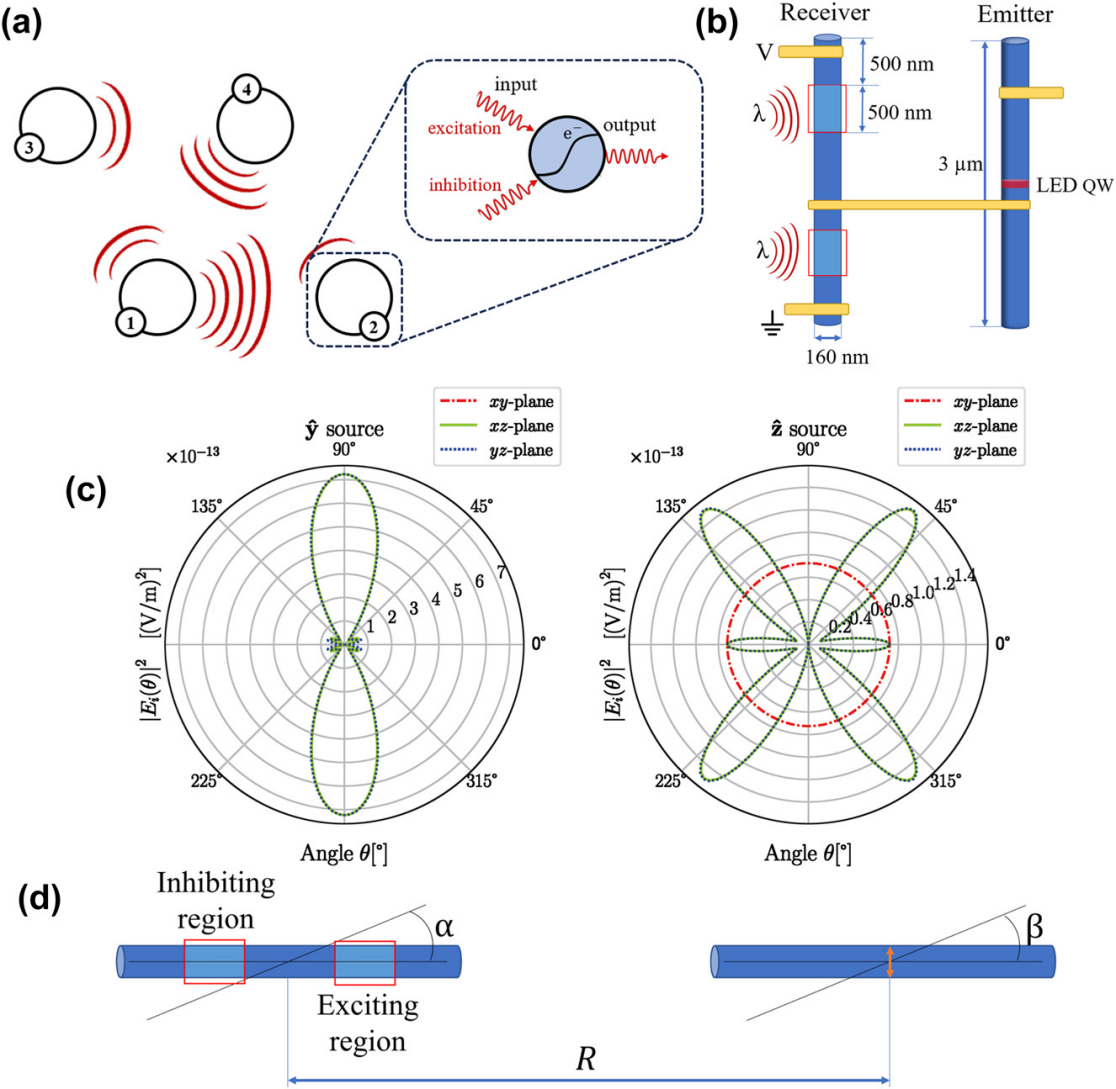
Illustrations of the main working principles of the neural network. (a) A schematic illustration of neural nodes communicating with each other via optical signals (b) Schematic two-NW device that can be used as receiver and emitter of optical signals achieving inhibiting and activating responses to light. Detailed circuit simulations are found in [16]. The red boxes represent photosensitive inhibiting/exciting regions of the receiver. Wavelengths used will depend on the III-V material combination. (c) Basic modelled emission spectra from a single emitter NW showing that emission will be from mainly the ends of the NW. The two plots are for different dipole sources placed in the centre of the wire. The *y* direction is along the long axis of the NW, while the *z* is perpendicular. (d) NW receiver/emitter design schematics for the simulations. It consists of a two-NW system where the receiver angle *α* is varied from 0 – 90 deg and emitter angle *β* −90° to 90°, while the distance is varied as *R* = 4, 5, 7, 9 µm.

In Figure 1a the basic network concept of our implementation is shown: Neural nodes emit light signals with varying strengths in different directions that can be picked up by other neural nodes with varying efficiency in a broadcasting scheme. A neural node will sum both inhibiting and exciting signals, and a light signal can be emitted based on a non-linear sigmoid function evaluation. Thus, the connectivity weights are given by the strength of the received light at each node. The individual neural node in our model is implemented via a two-NW device, one NW being the emitter for sending light signals to the other neural nodes and the other NW being the receiver for receiving the signals from other nodes. The light signals are evaluated electrically in the receiver NW with the desired “biological” sigmoid response to the exciting and inhibiting light signals (amplifying the input by ∼100 for fan-out [17]). The advantage of the III-V NW implementation is in the efficient energy conversion between photons and electrons and the possibility of implementation on Si. The detailed device function is discussed elsewhere [16], a schematic outline of a specific implementation of such a device is shown in Figure 1b. The emitter NW is 3 μm long and 160 nm wide cylindrical structure (Figure 1b) made from GaInAs with a quantum well LED in the centre of the NW that is made with Ga_*x*_In_1−*x*_P. By varying the material composition of the quantum well, the emitted light’s wavelength can be adjusted from 750 to 1,000 nm. A basic analytical modelling of the NW emission spectra is shown in Figure 1c, indicating that most of the signal is coming out of the ends of the NWs in the direction of their long axis. The emission from the quantum well LED in the middle of the NW can be assumed to be isotropic and can thus be seen as three dipoles (*x*, *y*, *z*) that are all emitting which leads to incoherent summation of the emission as opposed to lasers or single photon emission. This simplifies calculating the activation function by combining signals received from different neurons.

The geometry of the receiver NW is the same as the emitter NW, but instead of a quantum well LED, it has two phototransistors with one end of the NW connected to a power source and the other end to the ground (see Figure 1b). Shining light on the top end of the receiver NW will result in electron flow into the gate of the transistor controlling the NW LED – turning it on (excitation). Shining light on the bottom phototransistor will lead to electrons flowing from the gate into the ground turning off the LED (inhibition). Thus, we achieve a neural node function where inhibiting and exciting light signals on the receiver steer the (amplified) output light signal of the node via a sigmoid function. The photosensitive regions are 500 nm thick and placed symmetrically around the centre of the NW at 500 nm from the centre. Further details and simulations of the function can be found elsewhere [16], [17].

In this work, we concentrate on the simplest case where both exciting and inhibiting phototransistors work at the same wavelength. This simplifies the design of the NW. Working with a single wavelength for both phototransistors, the weights of the network are determined purely by the geometrical placement of the NWs. As a result, if we rotate the receiver NW 180°, the signal that used to excite the neuron would now be inhibiting it. This allows to achieve a large variability in weights by varying the rotation and position of the nodes. For each node we allow the NW emitter and receiver to have random angles with respect to each other while being placed with their centres on top of each other. Creating such a device is possible using advanced manufacturing techniques [22]. An even simpler alternative is placing the receiver and emitter nanowires parallel in each neuron, we have investigated this as well resulting in similar performance of the recurrent neural network described below.

The components of our optical neural network are placed inside a 500 nm thick Al_2_O_3_ quasi-2D waveguide to contain the light in the plane of the network lowering losses dramatically. The width of the waveguide is optimized so that the optical components in the middle of it are operating at its fundamental mode, ensuring the best conditions for a signal travelling to the other components. The optical components themselves will be placed at or near the plane of the waveguide. The waveguide itself is sandwiched between two 3 mm thick SiO_2_ substrates.

We simulate the optical interaction of one neuron’s emitter and another neuron’s receiver while changing the geometrical placement and orientation of both NWs (see SI). The simulations are done using Finite Difference Time Domain (FDTD) modelling implemented in Ansys Lumerical software. We recreate the quasi-2D Al_2_O_3_ waveguide and the GaInAs NWs as geometrical entities with realistic material properties.

The QW LED in the middle of the emitter NW is modelled as a combination of dipole sources. The dipole sources in the simulation emits light from 750 to 1,000 nm with 50 steps. To make a good representation of the average dipole we simulate 3 principal dipole directions (see SI, Fig. S.4) – and take the average of these three values. This approach assumes that the emission in the QW is isotropic. If the QW was anisotropic, we could use a more complex formula to calculate the average dipole than a simple average.

Both phototransistors of the receiver NW are simulated by placing a power absorption monitor in the appropriate locations. Because of the inhibiting and exciting nature of the phototransistors, the difference between these two monitor values is the weight of the neural connection. This means that the weight for any given geometrical placement of a NW receiver (with respect to the emitter NW) can be calculated with an equation:

(1) $$W=\frac{\sum_{i=1}^{3}\left(Pabs_{\mathrm{activator,}i}-Pabs_{\mathrm{inhibitor,}i}\right)}{3}$$

where *W* is the weight of a connection for a given geometrical placement, *Pabs* is the absolute absorbed power value from inhibiting and exciting monitors in the FDTD simulation, and *i* are the three principal dipole orientations in the centre of the emitter NW.

## 3 Network weight maps

To simulate the effects of different geometrical placements of the two NWs, we introduce the rotation angle *α* for the receiver NW, rotation angle *β* for the emitter NW and the parameter *R* for the distance between the centres of both NW. Since both NWs are in plane, by varying these three parameters we can achieve any geometrical placement of a two NW system.

**Figure 2:**
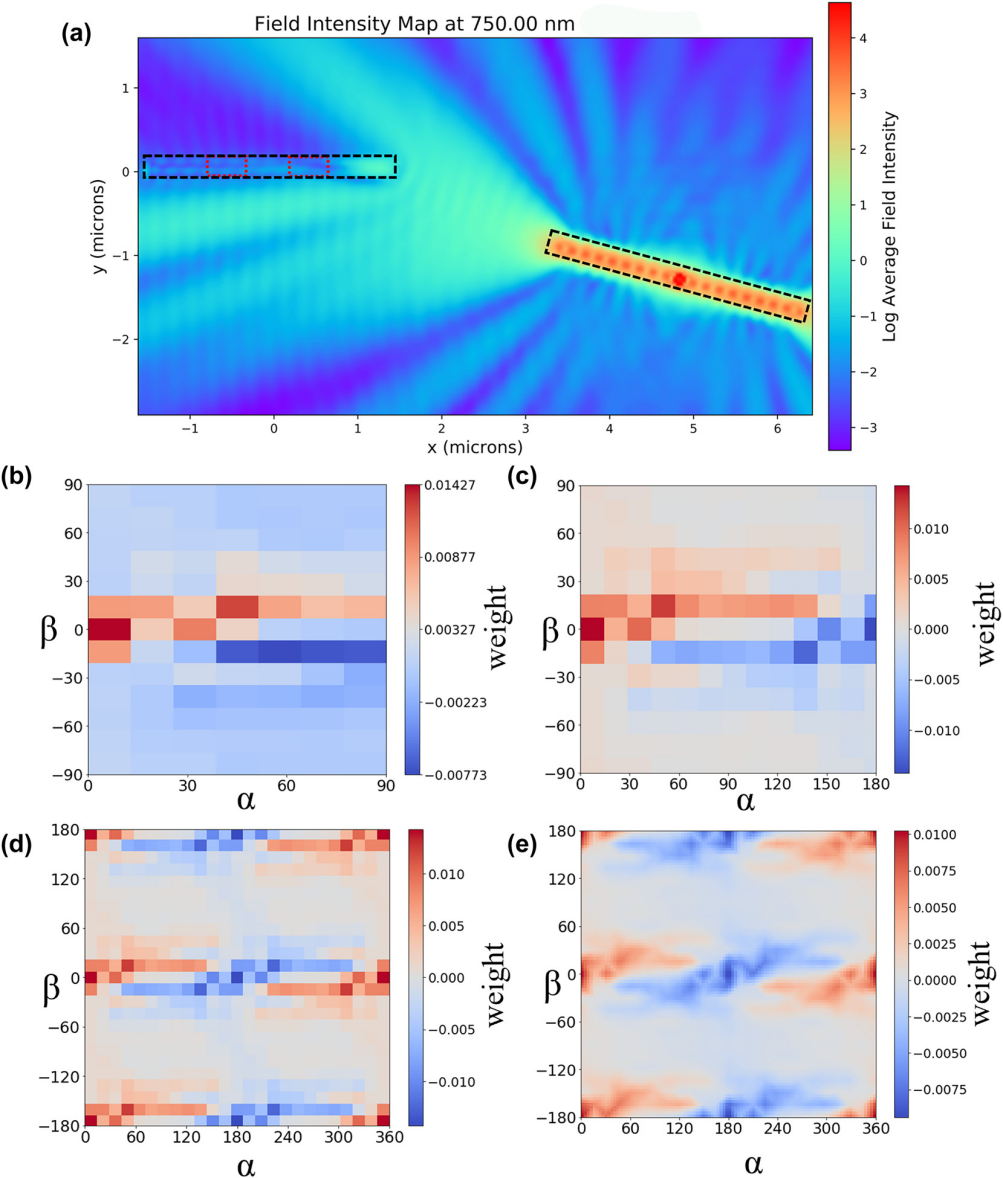
Electric field intensity around a NW emitter/receiver pair and the connection weight distributions at the receiver for different geometries. (a) An example of the field intensity map (average of 3 dipoles) for a geometrical placement with *α* = 15°, *β* = 0°, *R* = 5 µm. The diference between the two power absorption monitor values on the receiver NW (in red) creates a single point in later weight maps. (b) Example of a weight map (wavelength 750 nm and distance of 5 µm) for different rotation angles in the simulation region. (c) Extended simulation region using equation (2). (d) Extended weight data for full rotation angles in simulation data points. (e) Linearly interpolated weight map for all the rotation angles.

By exploiting the symmetry of the emitter NW and reverse symmetry of the receiver NW we can reduce the simulation region to −90 to 90° for *α* and 0–90° for *β*. We can get all the other rotations by extrapolating the data in the simulation region. The orientation with receiver angle *α* > 90 and emitter angle *β* is analogous to the receiver angle 180-*α* and emitter angle value 180-*β*, only the exciting and inhibiting monitors have changed places. This means that we can extend the original weight map (Figure 2b) in the receiver angle direction to 180° (Figure 2c) with the equation:

(2) $$W_{\alpha \in \left(90,180\right)}\left(\alpha ;\beta \right)=-W\left(180-\alpha ;180-\beta \right)$$

To find the weight values for the rest of the angles we can use the intrinsic symmetry of the emitter NW (the signal is precisely the same if we rotate the emitter NW 180°) and the intrinsic reverse symmetry of the receiver NW (if we rotate the receiver NW 180° the signal is equal in size but opposite in sign because the inhibiting and exciting regions have swapped places). This way we get the weights for all the rotation angles from 0 to 360° (Figure 2d).

We simulated the rotation angles with a 15-degree step and did the simulations at *R* = 4, 5, 7, 9 µm (full set of weight maps in the SI). Together with the three dipole directions for every geometrical placement, this amounts to over 1,000 simulations. To get the data for all the other rotation angles and distances in the simulated region *R* = 4–9 µm we use a linear interpolation function (result shown in Figure 2e) to get a weight value at any given geometrical placement.

From the 50 simulated wavelengths, we picked and later worked with three: 830 nm with the strongest overall signal, 750 nm with the most complex-looking weight map and 800 nm with the average-looking weight map (weight maps for all 50 wavelengths can be found in the SI).

From the weight maps in Figure 2 we observe that a wide variety of weights with both inhibiting and exciting nature can be achieved by varying the rotation angles of the NWs. This would indicate that complex network connectivity can be achieved already using rotation angles only.

**Figure 3:**
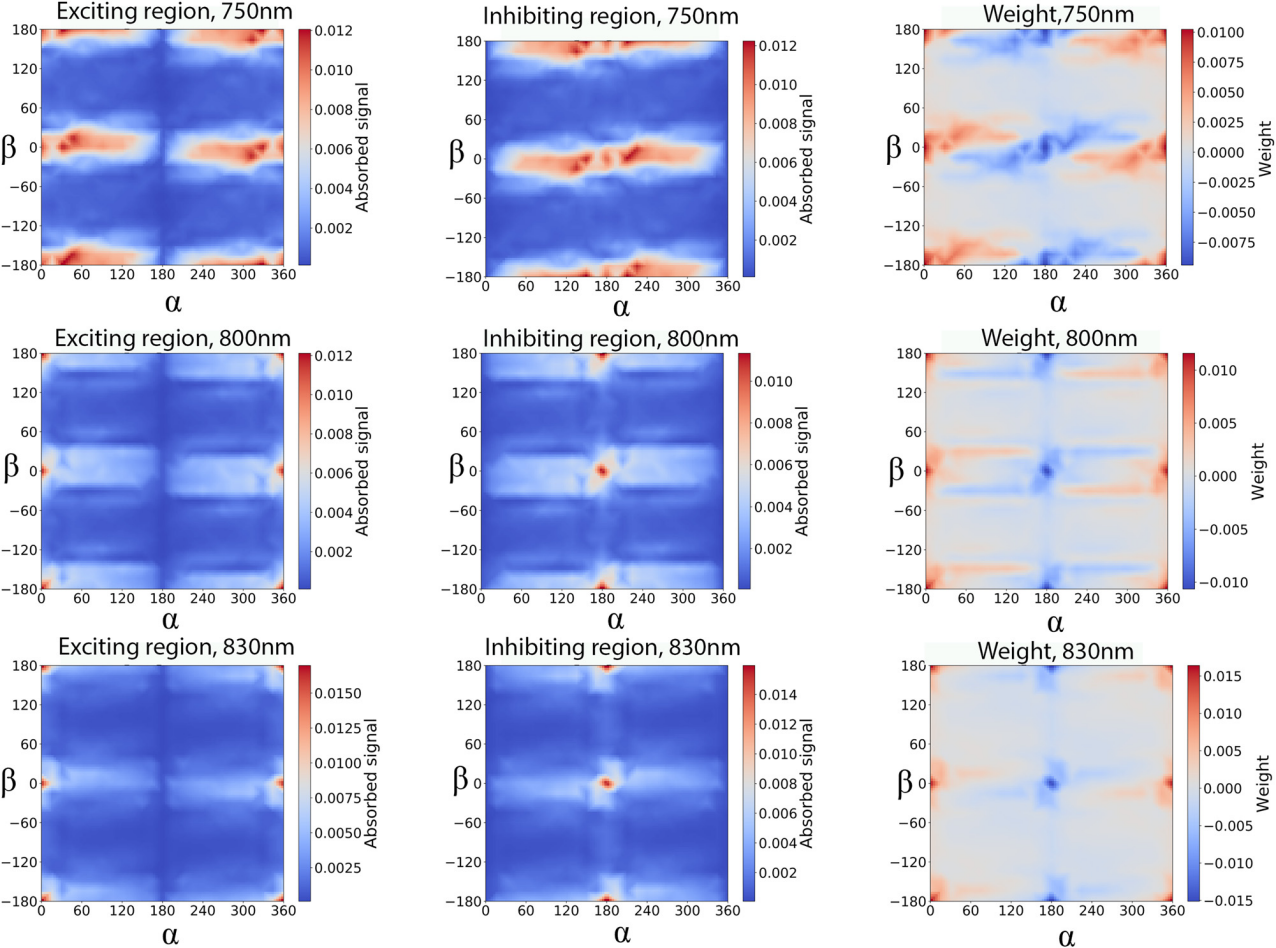
The signal strength (dipole average) based on absorbed light in the exciting region, inhibiting region of the nanowire receiver and the difference between the two (or the weight of neural connection) for 750, 800 and 830 nm with the distance between NWs of 5 µm.

To better demonstrate the working principle of the weight calculation, we also plot the exciting and inhibiting maps in Figure 3. We can see that the maps are practically identical, just one is shifted 180° in *α* direction. These maps show that a complex signal pattern can be achieved using geometrically induced weights only for the inhibiting signal. When we use the same wavelength for both exciting and inhibiting signals, we see a more complex weight map emerge. The positive values are where the exciting region absorbs more light, and the negative values are where the inhibiting region absorbs more light. We can see that the weight variation is spread over a larger angular range for 750 nm, which will allow access to more complex weight patterns when many wires are involved. The reason for the difference could be that it is 1/4th of the NW length, which is 3 μm, indicating that careful choice of the geometric parameters to match the wavelength is relevant to enable complex network designs.

The absolute sizes of the weights are in the range of 0.1–1%. This is consistent with the broadcasting concept in which signals are emitted in all directions in the 2D plane and only a fraction is picked up by each of the receivers (this is a basic property of a broadcasting scheme). To remedy this an amplification of ∼100 is built into the optical neural nodes. How much of the emitted light from each node is then used will depend on the number of receivers. However, previous work indicated that even the density as used here is highly energetically favourable compared to other types of hardware [16].

## 4 Reservoir neural network design and results

### 4.1 Network design

**Figure 4:**
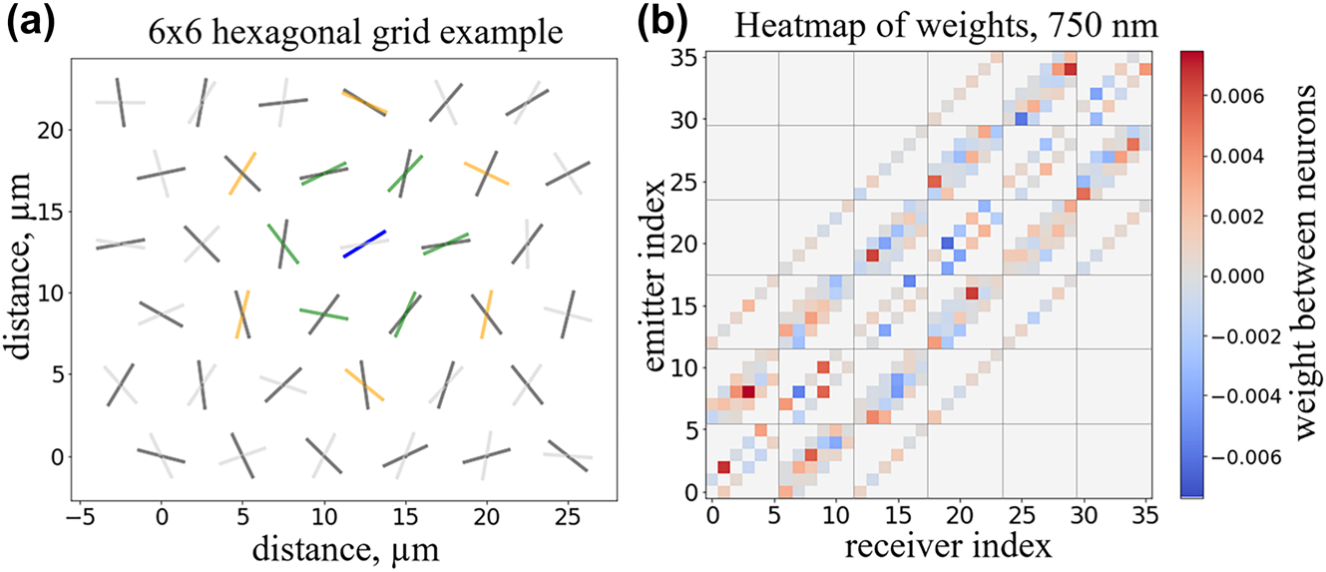
Representation of a 6 × 6 RNN and the resulting weight matrix. (a) An example of a hexagonal grid layout. One receiver NW is coloured blue and the emitter NWs of the first and second closest neighbours are coloured green and orange respectively. (b) An example of a weight matrix. The NW index number starts from 0 at the bottom left of the network and ends at the top right. The rows and columns of the grid are divided by lines on the map.

To test the usefulness of our concept, we implement it as a reservoir neural network (RNN). To create the RNN we placed the NWs in a hexagonal grid, where every grid point has a receiver and emitter NW. This way the receiver NW of each neuron gets a signal from the surrounding neurons’ emitter NW. The distance between hexagonal grid points is set to 5 µm. As the light signal falls of with distance (see SI) we only consider the first and second nearest neighbours in our model (Figure 4a). We set the self-excitation between the emitter and receiver in the same node to zero. As the NW primarily emits light out symmetrically from their ends and along the long axis of the NWs, the self-excitation between the emitter and receiver is negligible compared to the signal between surrounding neural nodes. The details and simulations confirming the validity of this assumption is found in Section 6 of the SI.

We calculate the weight matrix by first creating two matrices with random rotation angles for receiver and emitter NWs which define the orientation of every NWs in the grid. Then one by one we go through all the connections between the first and second closest neighbouring neurons (program code in the SI). We obtain the weight of a connection with an interpolation function from simulation data that takes three inputs – receiver rotation angle, emitter rotation angle and distance between the neurons – and gives out the weight of such a connection. The distance to the group of first closest neighbours is 5 µm and to the second closest neighbours it’s $5\sqrt{3}=8.66\;\mu m$ . With this method, we can create a weight matrix (Figure 4b) for any size of *N* × *M* where *N* is the number of rows and M is the number of columns in the hexagonal grid. From the weight matrix map in Figure 4b it can be concluded that a wide variation in connectivity weights are achievable. As the light is spread out from the individual emitters to many receivers, the weights are low, but the signals will subsequently be amplified in the nodes by a factor of ∼100 as discussed above. Signals could be further enhanced and more nodes reached by condensing the network, adding passive amplifiers or tailoring the medium between the wires.

For the RNN to operate, we also need to introduce the external input and output weights into our system. We suggest implementing the input weight by sending in the optical signal perpendicular to the plane of the network. This allows the best precision for selecting NW to excite and to control the power of excitation. Similarly, the output weights can be read as light emission perpendicular to the plane.

In this study we simulate two NWs at a time, ignoring influence of other wires on this communication as the absorption of the individual wire of the total amount of light is small. We focus on the optical properties of the NWs that make up the RNN (electrical function of the NWs was considered elsewhere [16], [17]). However, the presence of the electrical contacts powering the NW nodes can also affect the light propagation and thus the optical weights of the RNN. While not included in the present work, e.g. gold contacts would introduce further complexity in the signals due to scattering, which could benefit the RNNs function. Alternatively, indium tin oxide (ITO) transparent conductors could be used for the electrical contacts to limit the effects of the contacts or contacts could be placed in layers below the wires. However, generally adding structure to the medium surrounding the wires will open further opportunities to tailor the weights, thus increasing functionality.

### 4.2 Network characterization

In this study, we generated 100 unique permutations of rotation angle matrices for RNNs with sizes of 4 × 4, 6 × 6, 10 × 10, 20 × 20, and 32 × 32 (so from 16 to 1,024 neural nodes). Then we generated weight matrices for the operating wavelengths 750 nm, 800 nm and 830 nm for each of these neural networks. This approach systematically compares how varying the network size and the operating wavelength affects the neural network functionality.

Once the weight matrices of the RNN were ready, we trained their input and output weights, following the standard procedure for RNN, and applied them to a pattern recognition task. Each network was trained using the Mackey–Glass chaotic time series, a type of delay differential equation initially developed to model the delayed production and release of mature blood cells in a target population [23]. This data resembles signals encountered by neurons in biological systems, effectively enabling us to model their responses, which makes the Mackey–Glass series well-suited for training echo state networks (ESN). The training data and the ESN implementation protocol were sourced from the pyESN GitHub repository [24].

Training is done by teacher reinforcement training during periods *T*_train_ = 500 and 2000 data points, during which a training agent provides values of the Mackey–Glass time series as corrections to the ESN, gradually improving predictions over time. After the training period, the network is set free without the training agent for a similar duration of steps, and a normalised root mean square error (nrmse) value is calculated, given by

(3) $$\sigma_{\mathrm{nrmse}}=\frac{1}{\sigma_{\mathrm{target}}}\sqrt{\frac{\overset{n}{\underset{i=1}{\sum }}{\left(y_{\mathrm{target,}i}-y_{\mathrm{predicted,}i}\right)}^{2}}{n}}$$

to illustrate the cumulative difference between the target system, the Mackey–Glass time series, and the free-running ESN. *σ*_target_ is the standard deviation of the target system, *y*_target,i_ is the unitless values of the Mackey–Glass target system and the *y*_predicted,i_ is the ESN’s free running prediction respectively at time *i*, and *n* is the length of the predicted data. This value allows us to compare the functionality of the generated neural networks.

**Figure 5:**
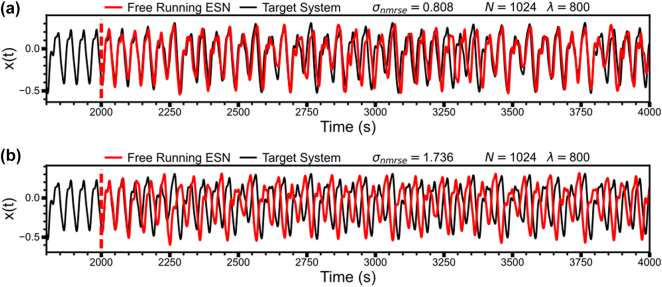
Examples of training of RNNa. Panels (a) and (b) illustrate the performance of two different 32 × 32 grid point RNNs trained for 2,000 steps, showing the difference in how well each free-running ESN predicts the Mackey–Glass time series, shown in both plots as the target system. The network in panel (a) has the lowest *σ*_nrmse_ out of all 32 × 32 grid point RNN simulated, whereas the network in panel (b) has the highest *σ*_nrmse_ value while still showing basic Mackey–Glass behaviour.

Lower values of *σ*_nrmse_ suggest a better performance of the NN within a free running time range. However, some exceptions remain, where the ESN would diverge a few points before the end of the prediction range. Target systems with *σ*_nrmse_ < 1 can be considered well-predicting of the specific time series, while networks in the range from *σ*_nrmse_ ∼ 1 − 2 behave like a Mackey–Glass system but predicts the specific sequence poorly. Results of a well predicting network compared to a non-deviating, but poorly predicting network are shown in Figure 5 for a network trained/predicting for 2000 steps. The predictive performance compared to the exact Mackey–Glass parameters between a network with *σ*_nrmse_ < 1compared to a network with *σ*_nrmse_ ≈ 2 vary significantly, as shown in Figure 5. Networks with *σ*_nrmse_ > 4 have diverged and no longer show a Mackey–Glass oscillatory behaviour.

**Figure 6:**
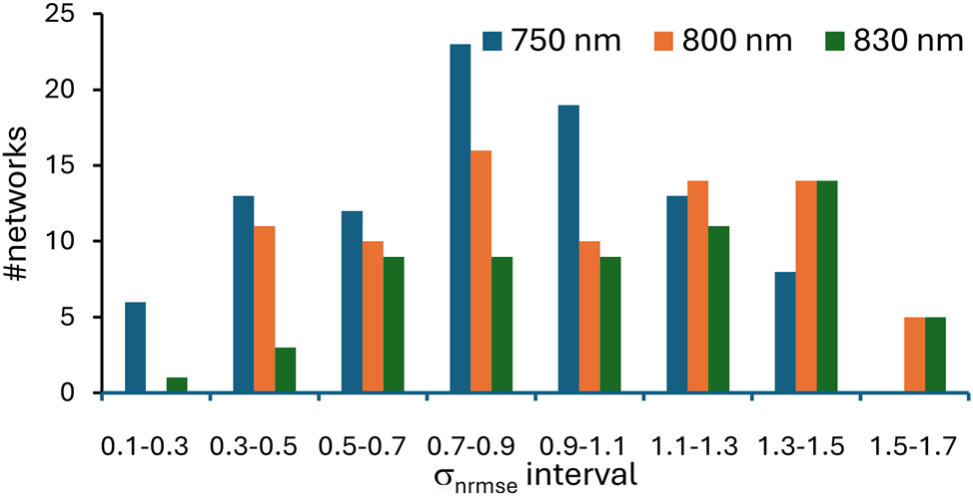
Histogram of simulated RNN networks within various *σ*_nrmse_ intervals after 500 training and 500 predictive steps for networks of *N* = 1,024. More successful networks can be found with the wavelength of 750 nm than for the two other wavelengths. For each wavelength 100 networks were simulated, but the histogram only shows networks with *σ*_nrmse_ < 2. The number of networks with higher *σ*_nrmse_ values than 2 were 6, 20, and 38 for 750, 800, 830 nm, respectively. This again indicate that best-performing networks are found with a wavelength of 750 nm.

From the substantial number of simulations performed, we provide statistics on how the RNN size and wavelength contribute towards the stability and performance of the network. Figure 6 show a histogram, for different ranges of *σ*_nrmse_ from 32 × 32 RNNs for the three different wavelengths for training/prediction of 500 steps. We can observe that the majority of RNNs behave as an oscillating Mackey–Glass system within the 500 data point prediction range. This range has previously been used for this type of prediction [21], [22] to estimate their reliability. For a number of physical ESN systems, a re-training is then needed at this point [21], [22]. In our case, a sizeable number of networks also retain predictive capabilities for 2000 steps, e.g. for 750 nm 17 % of the networks have a *σ*_nrmse_ < 1. See SI Table S3 for *σ*_nrmse_ values for all wavelengths and network sizes.

Increasing the size of the network improves the performance (see Table S3 in SI). Focusing on the 750 nm networks, the number of networks with *σ*_nrmse_ < 1 increase from 8 to 65 % by increasing the network size from 16 to 1,024 nodes. However, the functionality varies between the different networks even when just varying rotational angels. For networks using 800 nm and in particular 830 nm fewer networks have a function with *σ*_nrmse_ < 1 although an improvement for larger networks is also observed.

**Figure 7:**
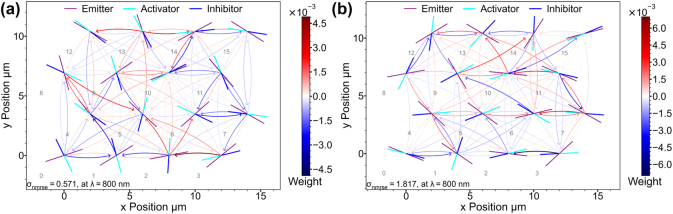
Panels (a) and (b) depict two 4 × 4 grid point RNNs with different random rotation angles for the NWs, evaluated at 750 nm with low and high *σ*_nrmse_ values, respectively. Weights generated in receivers from neighbouring emitters have been illustrated by arrows pointing from the emitter to the receiver. Only weights with absolute values > 0.01 are drawn, as to indicate only strongest connections. Both networks have many connections although their performance is markedly different. Numbering corresponds to receiver index shown in Figure 4b.

To illustrate the differing network performance, we compare an example of a good and a poorly performing network of small size (Figure 7). First it can be observed that both networks have a complex connectivity pattern indicating again that a wide range of complex networks are possible with our simple rotational weight concept yielding very different functionality. The reason for the markedly different behaviour is difficult to discern directly from the images. One difference is that in the poorly performing network, there are many circular connections between node pairs. We speculate that small self-reinforcing loops are created, which in turn degrades the overall predicting capabilities, as this type of network relies on complex node behaviour. 750 nm is systematically better than the other two wavelengths. That can be because of more broad distribution on the weight map (Figure 3) which can lead to more complex behaviour. However, for all three wavelengths complex connectivity patterns are found as well as networks with *σ*_nrmse_ < 1.

## 5 Conclusions

In conclusion, we find that an optical neural network made from III-V NWs relying on broadcasting and the near-field field distribution is enough to create complex neural network connections. We demonstrate that a RNN built with these components can perform the task of predicting a chaotic series. The III-V NWs make well predicting RNNs, where the weights of the network are purely passive – they are determined only by the geometrical placement and orientation of the NWs. These simple geometric variations can create a large connectivity weight space, which allows a wide variety of networks to be created. The variability is a consequence of the directional emission of the NWs along the long axis. But it is then the angle of the receiver that results in the weight variation, the emitter angle mainly determining if the emitter is communicating with a given receiver (it should point at it). Specific well-known types of III-V NWs were used for the simulation as benchmarks for their optoelectronic performance have previously been established. However, the basic concept explored here does not rely on the particular type of NWs but could be extended to a variety of materials and structures. For future analysis the mathematical equivalence to electrical driven networks is an interesting question to explore as considerable work been devoted to treating analog electrical neural networks. These mean field approaches can be used to understand the complex dynamics of such networks [25], [26], [27]. Developing the equivalence in optical systems is non-trivial [28], but represents an interesting research direction.

To fabricate networks of NWs in pre-determined patterns/orientations laterally on a substrate, several techniques exists: 1) Selective area growth (SAG) of NWs at predetermined locations by growth out of mask openings or using metal seed particles for lateral growth along the substrate [29], [30]. 2) Large scale multi-NW alignment is possible using liquid suspensions e.g. using electrical/chemical forces [29], fluid flow assembly [31], [32] or bubble-blow techniques [33]. These can be combined with dielectrophoretic methods to tailor local orientation and placement [34], [35]. 3) Manual placement and alignment approaches make use of various nanoprobe and micro-manipulator systems to move NWs from a growth substrate to a device substrate [36], [37]. Achievable translation and rotational precision vary among the different methods but can reach a similar level as the precision of the lithographic patterns used for guiding the placement or growth, which is ±10 nm for E-beam lithography [36]. Further, for the random networks the exact position of an individual NW does not matter so much, important is the ability to vary the orientation.

To increase the possible variation of weight distributions more complexity can be introduced in the system, for example creating holes in the quasi-2D waveguide will change the light propagation to steer the light to enhance or decrease a certain connection. A more complex emission pattern from the NWs, could result in further opportunities to tailor the weights which could be introduced via e.g. metallic antennas made during electric contact formation.

Another important extension would be to introduce memory for the neural network which could be done using of phase-changing, photochromatic or photorefractive materials for the quasi-2D waveguide [19], [38]. These materials change in response to light and then get back to their initial state, so these can be used for local neural network memories in the connections. Such synaptic systems should preferably work exclusively by responding to the light signal and with no further needs for electrical connections [19]. All-optical photoactive memories have been integrated on-chip with III-V NW components and their robustness tested showing a <0.6 % damage after 15,000 light spikes [19].

In the present paper we have explored the possibilities for creating networks operating at one wavelength. This is the most fundamental case where the weights arise purely from geometrical placement, but many expansions are possible, for example introducing the use of a different wavelength, polarization or other nanophotonic elements that can enhance or guide light signals. By varying the III-V material composition NWs can be sensitized to wide range of wavelengths [15]. Combining several photodiodes of different III-Vs in the same NW has been demonstrated [14] which could be employed to give exciting/inhibiting sections that operate at different wavelengths. Diodes across the range of III-V materials have been demonstrated in NWs, which could be used for creating networks that operate at multiple wavelengths [15]. In the future a 3D version with interconnected stacked layers of networks could be a further interesting option.

## Supplementary Material

Supplementary Material Details
