# Supplementary material for: Neural network connectivity by optical broadcasting between III-V nanowires
Source: Nanophotonics. 2025 Jul 4;14(15):2575–85. doi: 10.1515/nanoph-2025-0035 (PMC12322725; doi:10.1515/nanoph-2025-0035)
Supplement: Supplementary file 1 — Supplementary Material Details [file j_nanoph-2025-0035_suppl_001.pdf]

Kristians Draguns, Vidar Flodgren, David Winge, Alfredo Serafini, Aigars Atvars, Janis Alnis, Anders Mikkelsen*

Supplementary information (SI)

Neural network connectivity by optical broadcasting between III-V nanowires.

1 FDTD simulations details

First, we create a two nanowire (NW) system with parametrised geometry, so we can change the rotation of the NWs. The NWs are given by a cylinder with the length of 3 µm and diameter of 160 nm and they are placed in the same plane at distance R from each other. Then we place two 500 nm long power absorption monitors on one of the NWs, to represent the two photosensitive regions of receiver NW. The of these two power absorption monitors is the main result of these simulations. We place a dipole in the centre of the other NW to model the emitter. The FDTD simulations were carried out for 50 different wavelengths from 750 nm to 1 µm.

The Lumerical software does not allow to rotate a monitor, however since we are interested in rotating both NWs, we mimic the rotation of receiver NW by rotating the emitter NW around the centre of the receiver monitor Fig S.1.

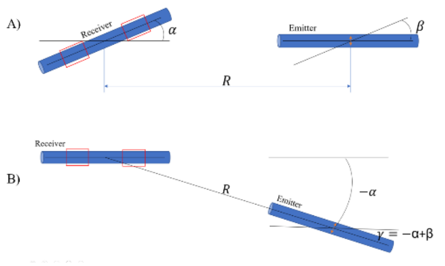

Fig. S.1: *A) A simple rotation of the receiver NW (impossible in Lumerical, because you can't rotate the monitors), B) The same rotation achieved with only manipulating the position of the emitter NW and dipole direction, so it stays fixed to the rotation of emitter NW. Since we only care about the relative position of the NWs, this is allowed.*

We place both NWs in the center of an Al_2O_3 waveguide, setting the perfectly matched layer boundary for the simulation inside the block of Al_2O_3, forming a quasi 2D waveguide.

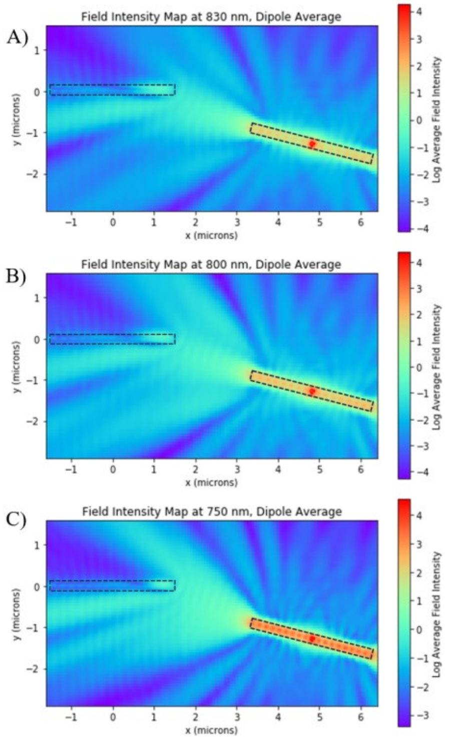

Fig. S.2: *Field intensity maps of the receiver and emitter NWs for A) 830 nm, B) 800 nm, C) 750 nm. Wires are indicated by dotted lines. Reciever is on the left and emitter on the right.*

*First author: University of Latvia, Riga, Latvia; kristians.draguns@lu.lv; 0000-0001-7357-3668
Second author: Lund University, Lund, Sweden, vidar.flodgren@sljus.lu.se; 0009-0006-8024-0259
Third author: Lund University, Lund, Sweden, winge.david@gmail.com; 0000-0001-5191-9728
Fourth author: Lund University, Lund, Sweden, alfredo.serafini@outlook.com; 0000-0003-0197-8935
Fifth author: University of Latvia, Riga, Latvia; Aigars.Atvars@lu.lv; 0000-0001-9933-0111
Sixth author: University of Latvia, Riga, Latvia; janis.alnis@lu.lv; 0000-0003-3824-9699
***Corresponding author (Seventh author):** Lund University, Lund, Sweden; anders.mikkelsen@sljus.lu.se

We perform simulations to determine the optimal height of the Al_2O_3waveguide, taking the height where the power absorbtion values for the NW comunication are maximum, ensuring that we operate in the mode of the waveguide. This corresponds to 500 nm thick Al_2O_3waveguide.

To better understand the field intensity in the simulation region, we made field intensity maps for the three wavelengths used to form neural networks as described in the main paper. In these maps we also take the dipole average as when calculating weight.

To better understand the dynamics of the signal propagation in the simulated system we also show field distribution images at different points in time to illustrate it.

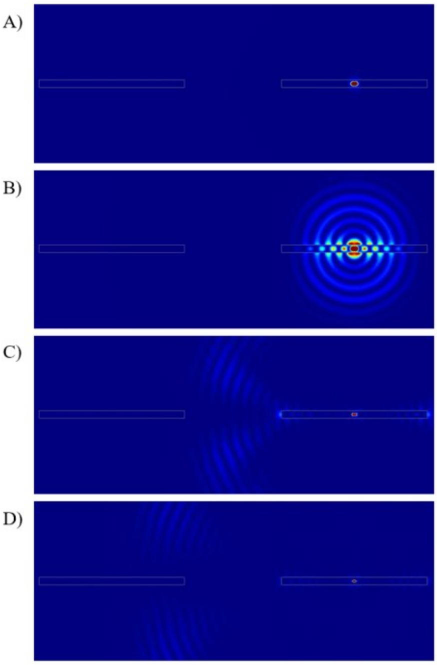

Fig. S.3: *The intensity of electric field for dipole 1 in different moments in time. A) The beginning of the light emission in the dipole inside the transmitter NW on the right. B) During the dipole oscillations, when the field is still close to the emitter NW, the field is almost symmetrical in all directions. C) After the oscillations have stopped, the field travels the medium and is concentrated in a cone shape. D) The field travels to the target monitors on the receiver NW on the left.*

2 Different dipoles

Weight maps were calculated separately for the emitter NW and the three different dipoles we combine (Fig. S.4).

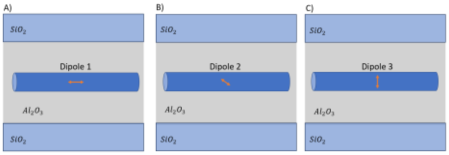

Fig. S.4: *Fig. S.4. The three principal dipole directions in the emitter NW LED QW. A) A dipole parallel to NW and 2D waveguide, B) a dipole perpendicular to NW and parallel to 2D waveguide, C) a dipole perpendicular to NW and 2D waveguide.*

All the dipoles are fixed in the reference frame of the emitter NW, so the emission profile of the NW itself does not change from the NW rotations. Dipole 1 is parallel to both the NW and the quasi 2D waveguide, dipole 2 is perpendicular to the NW but parallel to the waveguide, and dipole 3 is perpendicular to both the NW and the waveguide. These dipoles make the 3 principal dipole orientations, and every other dipole is a linear combination of these three. For the network we took simple average of these three dipoles, but more complex functions can be made if some of the dipoles are found to be more favoured than others.

In figures S.5-7 we show all the weight maps for different distances between NWs, different dipole orientations and different wavelengths. In figure S.8 we show all the calculated weight maps with dipole averages. From the weight maps we can see that the weight always decreases with increasing the distance and the weight map becomes more complex increasing the distance. We can also see that dipole 1 is always lower in intensity but has a more complex weight distribution than the other two dipoles. Dipoles 2 and 3 are close in intensity and the weight maps look similar, but the dipole 2 is always slightly more intensive. This is because the 2D waveguide interferes with the dipoles differently.

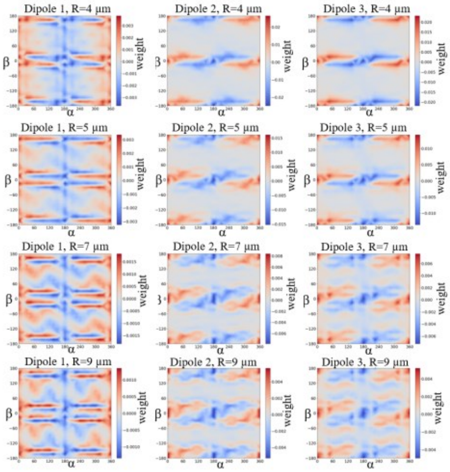

Fig. S.5: *The weight maps for full rotations of receiver NW angle and emitter NW angle for different dipole orientations and distances for 750nm wavelength. First row: distance between NW 4 microns, second row 5 microns, third row 7 microns, fourth row 9 microns. First column dipole orientation 1, second column dipole 2, third column dipole 3.*

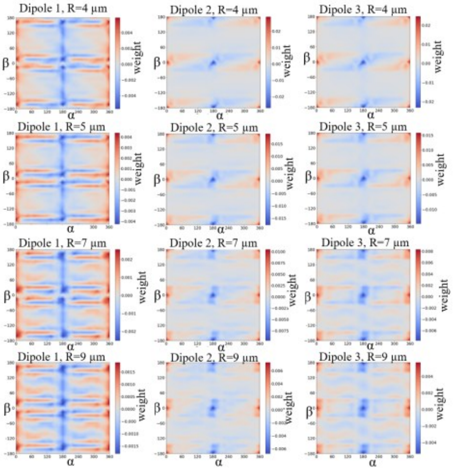

Fig. S.6: *The weight maps for full rotations of receiver NW angle and emitter NW angle for different dipole orientations and distances for 800nm wavelength. First row: distance between NW 4 microns, second row 5 microns, third row 7 microns, fourth row 9 microns. First column dipole orientation 1, second column dipole 2, third column dipole 3.*

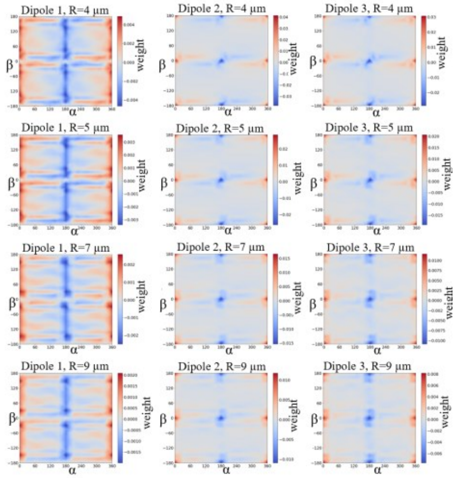

Fig. S.7: *The weight maps for full rotations of receiver NW angle and emitter NW angle for different dipole orientations and distances for 830nm wavelength. First row: distance between NW 4 microns, second row 5 microns, third row 7 microns, fourth row 9 microns. First column dipole orientation 1, second column dipole 2, third column dipole 3.*

3 All weight maps used in weight matrix creation

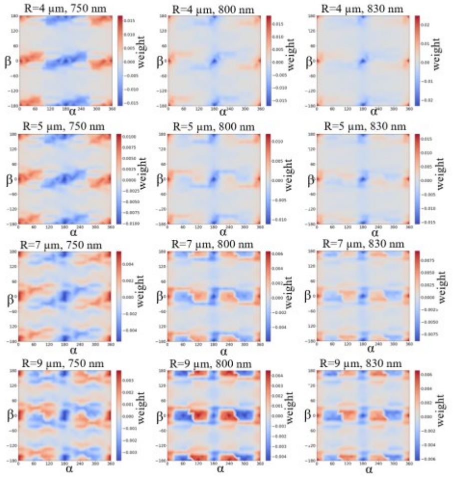

Fig. S.8: *The weight maps for full rotations of receiver NW angle and emitter NW angle for different distances and wavelength. First row: distance between NW 4 microns, second row 5 microns, third row 7 microns, fourth row 9 microns. First column wavelength 750nm, second column 800nm, third column 830nm.*

4 Weight map examples for all 50 wavelengths

The simulation was conducted using 50 equally spaced frequencies in the frequency domain. When these frequencies are converted to wavelengths, the resulting steps in the wavelength domain are non-uniform.

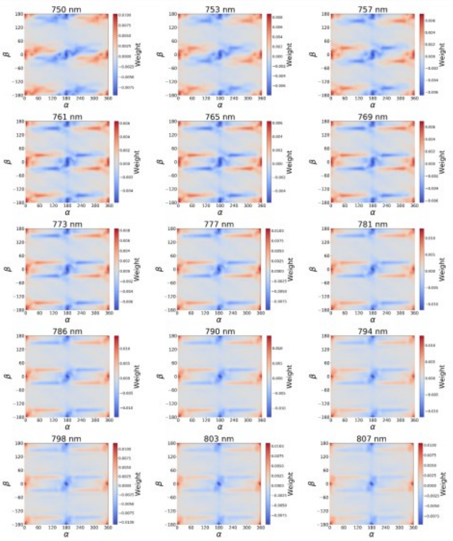

Fig. S.9: *Weight maps for all rotation angles for wavelengths from 750 nm to 807 nm at 5 µm distance between NWs.*

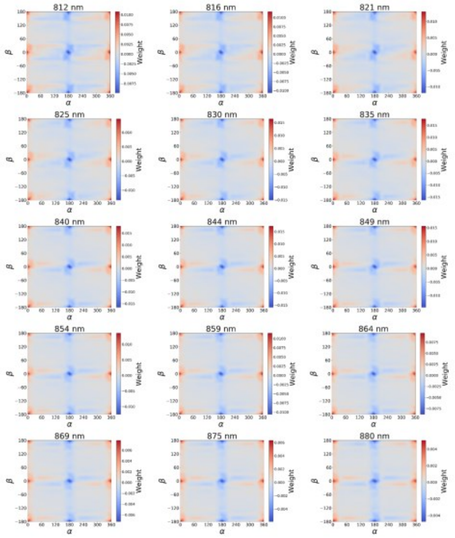

Fig. S.10: *Weight maps for all rotation angles for wavelengths from 812 nm to 880 nm at 5 µm distance between NWs.*

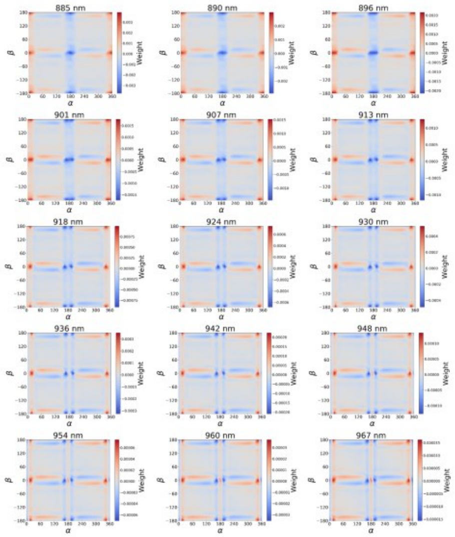

Fig. S.11: Weight maps for all rotation angles for wavelengths from 885 nm to 967 nm at 5 µm distance between NWs.

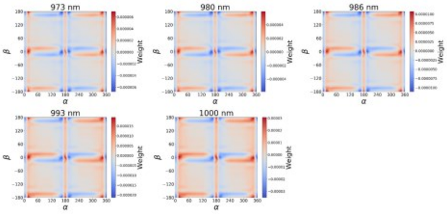

Fig. S.12: *Weight maps for all rotation angles for wavelengths from 973 nm to 1000 nm at 5 µm distance between NWs.*

5 Distance simulation

To understand the near field and far field effects of the two NW system, we created a simulation where we change the distance between NWs, taking the maximum weight achievable for a given distance between the NWs (NWs are parallel to each other, so rotation angels are zero) as seen in Fig.S.13. We can observe that the signal falls off monotonous in this range (note that <3 micron they will be physically touching) and observe that after 9 microns the signal has dropped by a factor of 10 compared the closest distance. This means that in a hexagonal reservoir network only the set of nearest and next nearest neighbours contributes significantly to the sum weight of the neural connection. All other weights in our model are set to zero.

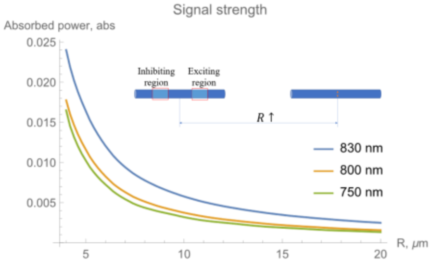

Fig. S.13: *Decreasing signal as a function of distance between the centres of the NWs with a schematic of the simulated NWs, both rotation angles set to zero.*

6 Self-excitation in a two NW neuron

In our simulations the receiver NW of one node and emitter NW of another node are placed in the centre-plane of the quasi-2D waveguide. When creating a network with the NWs placed in a hexagonal grid, where each grid point is a neuron with both a receiver and an emitter, this corresponds to both receiver and emitter NW sharing the same centre, overlapping each other. In reality the receivers would need to occupy one plane and the emitters another plane, both displaced from the centre plane of the waveguide in opposite directions.

Because the NW emits most light out from its ends along the long axis, the receiver of the same node will receive a weak signal even if they are close. However, the combined exciting/inhibiting signal is even weaker, since the emitter emits the light symmetrically on both sides and the photosensitive regions on the receiver NW are placed symmetrically as well, the self-excitations of the inhibiting and exciting monitors are equal and cancel each other out for the weight calculation for all rotation angles. We

provide a simulation of the transmitter and receiver NWs of the same neuron placed close together at 100 nm distance (Fig. S.14). We can see that while both monitors absorb some of the light, it is absorbed symmetrically and therefore doesn't introduce the self-excitation weight.

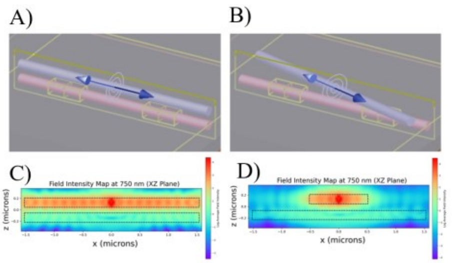

Fig. S.14: *A-B) Screenshots of Ansys Lumerical software with 3D placement and visible exciting and inhibiting monitors as well as the cut plane. C-D) Examples of field intensity maps for self-excitation of a neuron at 750 nm for parallel placement and 15-degree rotation with 100 nm between NWs.*

	750 nm	800 nm	830 nm
Pabs, Exciting	0.038173	0.035934	0.037830
Pabs, Inhibiting	0.038173	0.035934	0.037830
Weight	0	0	0

	750 nm	800 nm	830 nm
Pabs, Exciting	0.01843783	0.01740419	0.02575456
Pabs, Inhibiting	0.01843720	0.01740405	0.02575429
Weight	3.1666e-7	-5.8499e-7	1.3e-7

Table. S.1: *Tables with monitor values and weights for 750 nm, 800 nm and 830 nm for parallel and 15 degree rotation (top, bottom table respectively).*

In the main paper we focus on the case of perfectly placed NWs, where the self-excitation weight is close to zero for all rotation angles. The self-excitation in our system occurs only when a NW is placed away from the hexagonal grid point in some direction. This way the emitted light is no longer absorbed symmetrically on the receiver NW and the self-excitation weight arises. Misplacement of a NW would also change the weights with all the NW's neighbours, some of which will be closer, some further away. Such misplacement can be done intentionally to introduce self-excitation weight in the system. When creating a prototype, it is possible to ensure a NW placement of 10 – 50 nm accuracy, which we also simulated (Fig. S.15)

	750 nm	800 nm	830 nm
Parallel	0	0	0
Parallel, 10 nm offset	-0.001359	-0.000160	-0.001353
Parallel, 50 nm offset	0.001193	0.003513	0.000436
15-degree	3.1666e-7	-5.8499e-7	1.3e-7
15-degree, 10 nm offset	-0.00022	6.8551e-5	-5.2508e-5
15-degree, 50 nm offset	-0.00087	0.000389	-6.6573e-5

Table S.2: *A table with the calculated weights for 0 nm, 10 nm and 50 nm misplacement in x direction for parallel and 15-degree rotation placement.*

We can see that misplacement introduces some self-excitation weight, especially for parallel placement where the misplacement brings LED QW straightly closer to the exciting region. For 50 nm misplacement the self-excitation weight is noticeable compared to the max weight for each wavelength at 5 µm distance in fig.S.9-10, which amounts to 11.93%, 35.13% and 2.9% for 750 nm, 800 nm and 830 nm respectively. However, introducing a rotation angle the self-excitation weight drops to negligible values. This is because the emitter NW emits the light mainly in the direction it's pointing at (emission spectrum at fig. 1.C of the main text).

7 Result tables

The σ_{nrms} values for the simulated RNNs for predicting 500 and 2000 steps are summarized in a table to show how the different sizes and different operating wavelengths compare with each other (Fig.S.16).

8 Projecting the angles of NWs in hexagonal grid on the weight map

We start the formation of a network by creating two MxN matrices of random angles from 0 to 360 degrees – one for the receiver angles other for the emitter angles. These angles describe how the NWs are oriented in the network. For example the [1;1] element of each matrix represent the receiver and emitter angles for the bottom-left corner neuron in the hexagonal grid, and [M;N] element of each matrix represent the receiver and emitter angles for the top-right corner neuron in the hexagonal grid.

The function that maps the angles to a weight value f(R,α,β) is made for two NWs whose centres are on a horizontal line and the receiver NW is on the left and emitter NW is on the right, as in figure 1.C. To use this mapping function in a hexagonal grid we must take into account the extra angle that comes from the relative placement in the grid.

$$W_{x,y,x+1,y} = f(R, \alpha[x,y], \beta[x,y+1])$$

To use the weight formula for transmitter (x,y+2) and (x, y-2) we must subtract the orientation angle from both receiver angle and emitter angle and increase the distance by factor of $\sqrt{3}$, which is the distance to second closest neighbour in hexagonal grid.

$$W_{x,y,x,y+2} = f(R\sqrt{3}, \alpha[x,y] - 90, \beta[x,y+2] - 90)$$

$$W_{x,y,x,y-2} = f(R\sqrt{3}, \alpha[x,y] + 90, \beta[x,y-2] + 90)$$

When we calculate the weight for indexes on y+1 and y-1 row, we need to differentiate between odd and even rows. For example, the same relative orientation with grid angle 60 degrees on odd rows is for Δx=+1, Δy=+1, but on even rows it is for Δx=0, Δy=+1. This is because of the zig-zag edge of the hexagonal grid.

$$W_{x,y(odd),x+1,y+1} = f(R, \alpha[x,y] - 60, \beta[x+1,y+1] - 60)$$

$$W_{x,y(even),x,y+1} = f(R, \alpha[x,y] - 60, \beta[x,y+1] - 60)$$

Training 2000, prediction 2000 data points															
Size of NN	16 (4x4)			36 (6x6)			100 (10x10)			400 (20x20)			1024 (32x32)		
Wavelength	750	800	830	750	800	830	750	800	830	750	800	830	750	800	830
σ_{nrmse}<1	6%	1%	2%	1%	4%	2%	7%	4%	3%	15%	5%	4%	17%	10%	5%
σ_{nrmse}<2	89%	84%	90%	79%	84%	78%	71%	74%	82%	79%	70%	65%	86%	75%	40%
σ_{nrmse} ≥4	10%	14%	9%	21%	15%	20%	27%	25%	17%	20%	30%	35%	12%	24%	60%

Training 500, prediction 500 data points															
Size of NN	16 (4x4)			36 (6x6)			100 (10x10)			400 (20x20)			1024 (32x32)		
Wavelength	750	800	830	750	800	830	750	800	830	750	800	830	750	800	830
σ_{nrmse}<1	8%	7%	12%	10%	12%	9%	24%	21%	22%	14%	4%	5%	65%	41%	25%
σ_{nrmse}<2	90%	89%	96%	85%	88%	90%	75%	70%	83%	37%	22%	10%	94%	80%	62%
σ_{nrmse} ≥4	8%	10%	4%	14%	11%	9%	24%	28%	17%	62%	76%	90%	6%	20%	37%

Table. S.3: *Comparison of σ_{nrmse} values for all simulated RNNs (100 of each type). Top: Training/prediction for 2000 data points, Bottom: Training/prediction for 500 datapoints. As has been observed for other types of RNNS, networks are more successful for shorter prediction periods (see discussion in manuscript).*

In a similar way we calculate the all the other weights between a given receiver and the first and second closest neighbouring emitters by compensating the grid angle and taking into account the increased distance to second closest neighbours. Then we put it in a loop that goes through every receiver and calculates the weights for appropriate transmitters. On the edge of the network the receiver has fewer neighbours than in the middle, so the code has to know for which NW indexes to start and end the loop.

In summary we made a Wolfram Mathematica code that creates MxN receiver and emitter angle matrices, fills them with random angles, creates an empty MxNxMxN weight matrix, fills it with the appropriate calculated weights and transposes it to M*NxM*N wight matrix we use for neuron network analysis. The code investigates a single receiver and evaluates its surrounding emitters on different relative orientations numbered 1 to 12 depending on the relative grid angle. We generate 100 different weight matrices for the same network size, so we can qualitatively compare different network sizes and different wavelengths. In code the f1 function takes the interpolated data from simulations taking with arguments [R,α,β].

9 Wolfram Mathematica code for creating weight matrices

```
R = 5;
Mmax = 8;
Nmax = 8;
TA = Array[0, {Mmax, Nmax}];
RA = Array[0, {Mmax, Nmax}];
W = Array[0, {Mmax, Nmax, Mmax, Nmax}];


SeedRandom[1234];
(*
 For[z=0,z<100,z++{
SeedRandom[z];  *)

For[m = 0, m < Mmax, m++ {
  For[n = 0, n < Nmax, n++ {
    RA[[m, n]] = RandomReal[{0, 12}]*30;
    TA[[m, n]] = RandomReal[{0, 6}]*30;
    }]}]

For[m = 0, m < Mmax, m++ {
  For[n = 0, n < Nmax, n++ {
    For[j = 0, j < Mmax, j++ {
      For[k = 0, k < Nmax, k++ {
        W[[m, n, j, k]] = 0;
        }]}]}]}]

(* for emitter on point 1. *)
For[m = 0, m < Mmax, m++ {
  For[n = 0, n < Nmax - 1, n++ {
    W[[m, n, m, n + 1]] = f1[R, RA[[m, n]], TA[[m, n + 1]]];
    }]}]

(* for emitter on point 2. odd *)
For[m = 0, m < Mmax/2, m++ {
  For[k = 0, k < Nmax - 1, k++ {
    W[[2*m - 1, k, 2*m - 1 + 1, k + 1]] =
     f1[R*Sqrt[3], RA[[2*m - 1, k]] - 30,
      TA[[2*m - 1 + 1, k + 1]] - 30];
    }]}]

(* for emitter on point 2. even *)
For[m = 0, m < Mmax/2 - 1, m++ {
  For[k = 0, k < Nmax - 2, k++ {
    W[[2*m, k, 2*m + 1, k + 2]] =
     f1[R*Sqrt[3], RA[[2*m, k]] - 30, TA[[2*m + 1, k + 2]] - 30];
    }]}]

(* for emitter on point 3. odd *)
For[m = 0, m < Mmax/2, m++ {
  For[k = 0, k < Nmax, k++ {
    W[[m*2 - 1, k, 2*m - 1 + 1, k]] =
     f1[R, RA[[2*m - 1, k]] - 60, TA[[2*m - 1 + 1, k]] - 60];
    }]}]

(* for emitter on point 3. even *)
For[m = 0, m < Mmax/2 - 1, m++ {
  For[k = 0, k < Nmax - 1, k++ {
    W[[2*m, k, 2*m + 1, k + 1]] =
     f1[R, RA[[2*m, k]] - 60, TA[[2*m + 1, k + 1]] - 60];
    }]}]

(* for emitter on point 4. *)
For[m = 0, m < Mmax - 2, m++ {
  For[n = 0, n < Nmax, n++ {
    W[[m, n, m + 2, n]] =
     f1[R*Sqrt[3], RA[[m, n]] - 90, TA[[m + 2, n]] - 90];
    }]}]

(* for emitter on point 5. odd *)
For[m = 0, m < Mmax/2, m++ {
```

```
  For[k = 1, k < Nmax, k++ {
    W[[2*m - 1, k, 2*m - 1 + 1, k - 1]] =
      f1[R, RA[[2*m - 1, k]] - 120, TA[[2*m - 1 + 1, k - 1]] - 120];
    }]}]

(* for emitter on point 5. even *)
For[m = 0, m < Mmax/2 - 1, m++ {
  For[k = 0, k < Nmax, k++ {
    W[[2*m, k, 2*m + 1, k]] =
      f1[R, RA[[2*m, k]] - 120, TA[[2*m + 1, k]] - 120];
    }]}]

(* for emitter on point 6. odd *)
For[m = 0, m < Mmax/2, m++ {
  For[k = 2, k < Nmax, k++ {
    W[[2*m - 1, k, 2*m - 1 + 1, k - 2]] =
      f1[R*Sqrt[3], RA[[2*m - 1, k]] - 150,
       TA[[2*m - 1 + 1, k - 2]] - 150];
    }]}]

(* for emitter on point 6. even *)
For[m = 0, m < Mmax/2 - 1, m++ {
  For[k = 1, k < Nmax, k++ {
    W[[2*m, k, 2*m + 1, k - 1]] =
      f1[R*Sqrt[3], RA[[2*m, k]] - 150, TA[[2*m + 1, k - 1]] - 150];
    }]}]

(* for emitter on point 7.  *)
For[m = 0, m < Mmax, m++ {
  For[n = 1, n < Nmax, n++ {
    W[[m, n, m, n - 1]] =
      f1[R, RA[[m, n]] - 180, TA[[m, n - 1]] - 180];
    }]}]

(* for emitter on point 8. odd *)
For[m = 1, m < Mmax/2, m++ {
  For[k = 2, k < Nmax, k++ {
    W[[2*m - 1, k, 2*m - 1 - 1, k - 2]] =
      f1[R*Sqrt[3], RA[[2*m - 1, k]] - 210,
       TA[[2*m - 1 - 1, k - 2]] - 210];
    }]}]

(* for emitter on point 8. even *)
For[m = 0, m < Mmax/2, m++ {
  For[k = 1, k < Nmax, k++ {
    W[[2*m, k, 2*m - 1, k - 1]] =
      f1[R*Sqrt[3], RA[[2*m, k]] - 210, TA[[2*m - 1, k - 1]] - 210];
    }]}]

(* for emitter on point 9. odd *)
For[m = 1, m < Mmax/2, m++ {
  For[k = 1, k < Nmax, k++ {
    W[[2*m - 1, k, 2*m - 1 - 1, k - 1]] =
      f1[R, RA[[2*m - 1, k]] - 240, TA[[2*m - 1 - 1, k - 1]] - 240];
    }]}]

(* for emitter on point 9. even *)
For[m = 0, m < Mmax/2, m++ {
  For[k = 0, k < Nmax, k++ {
    W[[2*m, k, 2*m - 1, k]] =
      f1[R, RA[[2*m, k]] - 240, TA[[2*m - 1, k]] - 240];
    }]}]

(* for emitter on point 10. *)
For[m = 2, m < Mmax, m++ {
  For[n = 0, n < Nmax, n++ {
    W[[m, n, m - 2, n]] =
      f1[R*Sqrt[3], RA[[m, n]] - 270, TA[[m - 2, n]] + 90];
    }]}]

(* for emitter on point 11. odd *)
For[m = 1, m < Mmax/2, m++ {
  For[k = 0, k < Nmax, k++ {
    W[[2*m - 1, k, 2*m - 1 - 1, k]] =
      f1[R, RA[[2*m - 1, k]] - 300, TA[[2*m - 1 - 1, k]] + 60];
    }]}]

(* for emitter on point 11. even *)
For[m = 0, m < Mmax/2, m++ {
  For[k = 0, k < Nmax - 1, k++ {
    W[[2*m, k, 2*m - 1, k + 1]] =
      f1[R, RA[[2*m, k]] - 300, TA[[2*m - 1, k + 1]] + 60];
    }]}]

(* for emitter on point 12. odd *)
For[m = 1, m < Mmax/2, m++ {
  For[k = 0, k < Nmax - 1, k++ {
    W[[2*m - 1, k, 2*m - 1 - 1, k + 1]] =
      f1[R*Sqrt[3], RA[[2*m - 1, k]] - 330,
       TA[[2*m - 1 - 1, k + 1]] + 30];
    }]}]

(* for emitter on point 12. even *)
For[m = 0, m < Mmax/2, m++ {
  For[k = 0, k < Nmax - 2, k++ {
    W[[2*m, k, 2*m - 1, k + 2]] =
      f1[R*Sqrt[3], RA[[2*m, k]] - 330, TA[[2*m - 1, k + 2]] + 30];
    }]}]


W1 = ArrayReshape[W, {Mmax*Nmax, Mmax*Nmax}];
TableForm[W1]
```
